# Distinct Patterns of Brain Metabolism in Patients at Risk of Sudden Unexpected Death in Epilepsy

**DOI:** 10.3389/fneur.2021.623358

**Published:** 2021-11-24

**Authors:** Benjamin P. Whatley, Joel S. Winston, Luke A. Allen, Sjoerd B. Vos, Ashwani Jha, Catherine A. Scott, April-Louise Smith, Fahmida A. Chowdhury, Jamshed B. Bomanji, Samden D. Lhatoo, Ronald M. Harper, Beate Diehl

**Affiliations:** ^1^Department of Clinical and Experimental Epilepsy, UCL Queen Square Institute of Neurology, London, United Kingdom; ^2^Division of Neurology, Dalhousie University, Halifax, NS, Canada; ^3^Department of Clinical Neurophysiology, National Hospital for Neurology and Neurosurgery, London, United Kingdom; ^4^Wellcome Trust Centre for Human Neuroimaging, UCL Queen Square Institute of Neurology, London, United Kingdom; ^5^Department of Basic and Clinical Neuroscience, Institute of Psychiatry, Psychology and Neuroscience, King's College London, London, United Kingdom; ^6^Department of Clinical Neurophysiology, King's College Hospital, London, United Kingdom; ^7^Epilepsy Society MRI Unit, Chalfont St Peter, United Kingdom; ^8^The Center for SUDEP Research, National Institutes of Neurological Disorders and Stroke, Bethesda, MD, United States; ^9^Neuroradiological Academic Unit, Queen Square Institute of Neurology, University College London, London, United Kingdom; ^10^Centre for Medical Image Computing, University College London, London, United Kingdom; ^11^Department of Brain Repair and Rehabilitation, UCL Queen Square Institute of Neurology, London, United Kingdom; ^12^Institute of Nuclear Medicine, University College London, London, United Kingdom; ^13^Epilepsy Center, Neurological Institute, University Hospitals Case Medical Center, Cleveland, OH, United States; ^14^Department of Neurology, University of Texas Health Sciences Center at Houston, Houston, TX, United States; ^15^Brain Research Institute, University of California, Los Angeles, Los Angeles, CA, United States; ^16^Department of Neurobiology, David Geffen School of Medicine at University of California, Los Angeles, Los Angeles, CA, United States

**Keywords:** SUDEP, positron emission tomography, central autonomic regulation, cardiorespiratory regulation, epilepsy, bilateral tonic-clonic seizure

## Abstract

**Objective:** To characterize regional brain metabolic differences in patients at high risk of sudden unexpected death in epilepsy (SUDEP), using fluorine-18-fluorodeoxyglucose positron emission tomography (^18^FDG-PET).

**Methods:** We studied patients with refractory focal epilepsy at high (*n* = 56) and low (*n* = 69) risk of SUDEP who underwent interictal ^18^FDG-PET as part of their pre-surgical evaluation. Binary SUDEP risk was ascertained by thresholding frequency of focal to bilateral tonic-clonic seizures (FBTCS). A whole brain analysis was employed to explore regional differences in interictal metabolic patterns. We contrasted these findings with regional brain metabolism more directly related to frequency of FBTCS.

**Results:** Regions associated with cardiorespiratory and somatomotor regulation differed in interictal metabolism. In patients at relatively high risk of SUDEP, fluorodeoxyglucose (FDG) uptake was increased in the basal ganglia, ventral diencephalon, midbrain, pons, and deep cerebellar nuclei; uptake was decreased in the left planum temporale. These patterns were distinct from the effect of FBTCS frequency, where increasing frequency was associated with decreased uptake in bilateral medial superior frontal gyri, extending into the left dorsal anterior cingulate cortex.

**Significance:** Regions critical to cardiorespiratory and somatomotor regulation and to recovery from vital challenges show altered interictal metabolic activity in patients with frequent FBTCS considered to be at relatively high-risk of SUDEP, and shed light on the processes that may predispose patients to SUDEP.

## Introduction

Sudden unexpected death in epilepsy (SUDEP) is the most common cause of premature death in people with epilepsy, and is second only to stroke as a neurological cause of years of life lost in the general population (1). Patients with medically-refractory epilepsy and convulsive seizures are at particularly high risk (2).

The underlying mechanisms of SUDEP are unclear, but a landmark study of physiological changes immediately prior to SUDEP identified a consistent pattern of cardiorespiratory collapse: a bilateral tonic-clonic seizure is followed by a short, variable period of normal cardiovascular and respiratory patterning, and then a combination of central apnoea and bradycardia, ultimately evolving to asystole (3). The absence of intrinsic cardiac pathology or lung disease in these patients points to a failure of central regulatory processes controlling these vital functions, and there is likely overlap with brain areas implicated in other conditions associated with sudden death, such as congenital central hypoventilation syndrome (CCHS) (4), sudden infant death syndrome (5), and heart failure (6).

Biomarkers for SUDEP risk are urgently needed. Sudden unexpected death in epilepsy is a tragic outcome in epilepsy, but sudden death remains sufficiently rare that it is difficult to conduct prospective mechanistic studies in this population. One option is to use a surrogate marker of risk to stratify patients, but no validated risk prediction tools exist. Several SUDEP risk factors have been identified, with frequent convulsive seizures, anti-seizure polytherapy, and increased duration of epilepsy emerging as the strongest predictors (7). However, two risk assessment inventories, the SUDEP-7 (8) and the International League Against Epilepsy (ILAE) combined analysis score (9), fail to distinguish between individuals dying with SUDEP and living patient controls with epilepsy. A recently validated probabilistic prediction score seems promising but is clinically-based and not designed to provide mechanistic insight (10).

Patients who have died of SUDEP demonstrate changes in brain volumes within regions that are key for regulating breathing and cardiovascular functions (11, 12). In these patients, loss of gray matter in both medial and lateral cerebellum, periaqueductal gray (PAG), left medial and posterior thalamus, left hippocampus, and posterior cingulate was demonstrated using retrospective analysis of existent MRI scans. These volume losses are significant, but less marked in patients at high risk of SUDEP, as defined by three or more generalized tonic-clonic seizures (GTCS) per year, and were absent in those patients without GTCS. Gray matter volumes in limbic areas and a subcallosal region (BA25) were increased in patients with high risk and those who died of SUDEP (11, 12). Moreover, functional connectivity between brain sites that regulate breathing and cardiovascular control (including thalamus, brainstem, anterior cingulate, putamen, amygdala, medial/orbitofrontal cortex, insula, hippocampus, caudate, and subcallosal cortex) are affected in patients at high risk of SUDEP (13, 14). We reasoned that interictal metabolic changes within those regions would provide distinct and complementary information about the neurobiological processes mediating SUDEP risk. We therefore analyzed fluorine-18-fluorodeoxyglucose (^18^FDG) uptake in patients at high and low risk of SUDEP, and sought to identify regions that demonstrate metabolic differences between these groups.

## Methods

### Study Design and Patient Selection

We enrolled 135 patients aged >18 years with medically refractory focal epilepsy who were being evaluated in the video telemetry unit at the National Hospital for Neurology and Neurosurgery, London, UK, and who had undergone fluorine-18-fluorodeoxyglucose positron emission tomography (^18^FDG-PET) scans as part of their pre-surgical workup. Patients were excluded if original PET data could not be retrieved, if there were significant structural abnormalities seen on MRI, or if there were missing demographic or clinical data critical to the analyses. Patients enrolled between April 2015 and December 2018 provided written informed consent to participate in a prospective study evaluating autonomic, respiratory, and imaging biomarkers of SUDEP. This study was approved by the relevant ethics committees (14/SW/0021 and 19/SW/0071 South West-Central Bristol Ethics Committee).

Frequency of GTCS has emerged as the single most-important risk factor for SUDEP, and the largest increase in risk occurs with three or more of these seizures per year (7). We therefore chose to classify our patients using a binary SUDEP risk score, according to focal to bilateral tonic-clonic seizures (FBTCS) frequency in our group of patients with intractable focal epilepsy undergoing PET for pre-surgical evaluation ≥3 FBTCS per year “high risk,” *n* = 56) vs. zero FBTCS per year “low risk,” *n* = 69)]. In our previous studies, this approach distinguished >80% of SUDEP cases (11). The screening led to the exclusion of 10 patients at intermediate risk. We used this cutoff as a conservative marker of SUDEP risk, as having ≥3 FBTCS per year confers the highest risk of SUDEP. Sudden Unexpected Death in Epilepsy is a GTCS-related event (3, 15), and therefore the low risk group was restricted to those with zero FBTCS in the previous year.

All clinical data used for risk assessment and/or as covariates were extracted from multidisciplinary team meeting notes via chart review, and from a local database capturing autonomic, respiratory, and imaging biomarkers of SUDEP. Mann-Whitney-*U* and Chi-squared tests were used to compare demographic, clinical, and PET signal characteristics across the high- and low-risk groups, implemented in MATLAB 2018b (Mathworks).

### PET Acquisition Parameters

Patients were asked to fast for a minimum of six hours prior to scanning. All scans were acquired at the Institute of Nuclear Medicine, University College London Hospitals, on either a Discovery VCT (140 kV, 80 mA, 0.8 s) or Discovery 710 PET/CT (120 kV, 170 mA, 0.8 s) (GE Healthcare). Patients were injected with a target activity of 250 MBq and an uptake time of 30–45 min. Scans were acquired for 15 min per bed (slice thickness: 3.27 mm) and reconstructed (3 iterations, 20 subsets, post-filtering: Hanning, 4 mm). Time-of-flight imaging was used for those scans acquired on the GE Discovery 710. Reconstructed images were exported as DICOM files, and converted to NifTi format for further pre-processing.

### Image Processing and Statistics—Voxel-Wise Analysis

Images were processed and analyzed using SPM12 (Wellcome Center for Human Neuroimaging) implemented in MATLAB 2018b (Mathworks). Details of our adapted voxel-based morphometry methodology (16) have previously been published (17). Individual PET scans were non-linearly normalized to an FDG-PET template in ICBM/MNI space that has been validated in a dementia cohort in the presence of brain atrophy (18, 19). Image intensity was modulated during normalization to account for non-linear spatial warping during modulation. Normalized images were resliced to 3 mm isotropic voxels.

We were primarily interested in the gray matter signal; thus, a binary optimal-threshold mask was created to include only consistently high-intensity voxels in the spatially-normalized scans. Compared to the standard SPM method for creating binary masks, this strategy minimizes the risk of excluding regions with very low intensity that may bear some physiological relevance, particularly in the context of brain atrophy (20). This mask was then applied to all spatially-normalized ^18^FDG-PET images. As the final step prior to statistical analysis, images were smoothed with a 6 mm FWHM Gaussian filter.

Several scanning and patient factors can introduce between-subject signal variation in ^18^FDG-PET images, many of which have no physiological bearing. These factors include the individual pharmacodynamics of the ^18^FDG levels, and the timing and dosage of ^18^FDG relative to the imaging acquisition. To account for these variations, we estimated the background ^18^FDG-PET signal per scan for later use in our statistical models, using voxel intensities extracted from high-intensity areas, ventricular compartment, and white matter compartment. High-intensity activity was indexed as the summed intensities of all raw ^18^FDG-PET counts within the optimal-threshold mask, ventricular and white matter activity as the summed intensities of the voxels falling within the CSF and white matter tissue compartments, respectively (based on the Neuromorphometrics atlas included with SPM).

Statistical parametric mapping was used to explore whole brain differences in ^18^FDG uptake between the groups (21, 22). Sudden Unexpected Death in Epilepsy risk (the binary covariate of interest) was entered as an independent variable into a general linear model, with additional covariates to control for confounding including age, sex, and weight, and total high-intensity activity, white matter activity and CSF intensity. A planned *t*-test was used to estimate the effect of SUDEP risk, the effect of interest. In keeping with standard methodology, the resultant map of *t*-statistics was first thresholded at a cluster-forming threshold of *p* < 0.001. Then, the cluster-level threshold for statistical significance was set at *p* < 0.05 after family-wise error correction for multiple comparisons across all brain voxels. Figures were thresholded at *p* < 0.001 uncorrected, with a cluster extent threshold of *k* = 110 voxels to aid visualization.

### Image Analysis—Role of FBTCS Frequency

Given that our binary SUDEP risk score was based on FBTCS frequency threshold, we explored whether regional differences in ^18^FDG uptake were more directly related to convulsion frequency rather than our surrogate score for SUDEP risk. This analysis was undertaken within the 63 participants who had at least one FBTCS in the previous year. FBTCS frequency was log_10_-transformed to obtain a normal distribution and entered into a general linear model with similar additional confounding covariates as the primary SUDEP risk analysis. The effect of interest, FBTCS frequency, was estimated with a planned *t*-test, and statistical thresholds were set as previously described.

## Results

### Patient Characteristics

High- and low-risk SUDEP groups did not differ significantly for age, sex, weight, height, duration of epilepsy, number of anti-epileptic drugs, epilepsy duration, frequency of all seizure types, presence of MRI lesions, or final nuclear medicine impression of the PET scans (Table 1). Measures used to estimate the background signal of the PET scans did not significantly differ between the two risk groups (Table 2).

**Table 1:** Group characteristics of patients with high and low SUDEP risk.

	**Low risk** **(*n* = 69)**	**High risk** **(*n* = 56)**	***p***
Age, years, median, IQR	33.2, 23.5–41.1	31.1, 26.3–37.5	0.51^a^
Sex, M:F	35:34	33:23	0.36^b^
Weight, kg, median, IQR	75.0, 65.0–89.2	78.5, 67.5–95.5	0.27^a^
Height, m, median, IQR	1.70, 1.63–1.80	1.73, 1.64–1.81	0.36^a^
Epilepsy duration, years, median, IQR	17.5, 12.0–26.6	16.9, 9.8–25.2	0.50^a^
Number of AEDs, median, IQR	3, 2–3	3, 2–3	0.39^a^
Frequency of FBTCS (per year), median, IQR	0, 0	24, 12–52	–
Frequency of all seizure types (per year), median, IQR	208.0, 78.0–527.8	243.0, 116.5–525.0	0.50^a^
MRI lesion, yes:no	16:53	14:42	0.81^b^
PET results (normal/temporal/other)	21:39:9	10:30:16	0.06^b^

*IQR, interquartile range*.

a *Mann-Whitney-U*.

b *Pearson chi-square*.

**Table 2:** Estimates of background PET signal.

	**Low risk** **(*n* = 69)**	**High Risk** **(*n* = 56)**	***p***
Sum of high intensity voxel intensities (median, IQR, × 10^8^)	7.18, 5.72–8.63	6.95, 5.99–8.35	0.53^a^
Sum of ventricular compartment voxel intensities (median, IQR, × 10^6^)	6.10, 4.93–7.93	5.99, 4.69–7.23	0.53^a^
Sum of white matter compartment voxel intensities (median, IQR, × 10^8^)	1.89, 1.52–2.33	1.83, 1.57–2.18	0.49^a^

*IQR, interquartile range*.

a *Mann-Whitney-U*.

### Altered Glucose Uptake in High vs. Low SUDEP Risk Subjects

**Figure 1:**
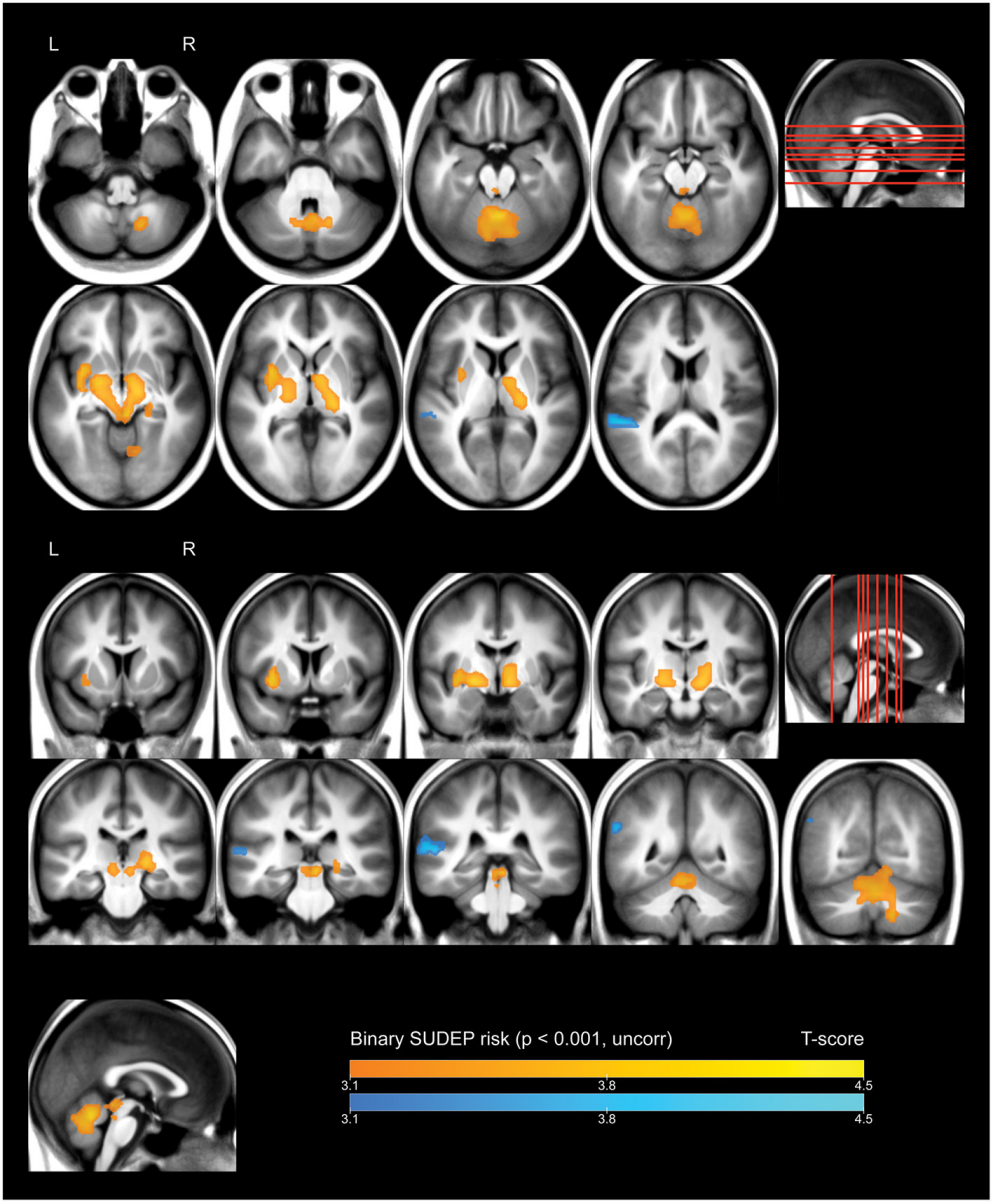
Whole brain SPM results, high vs. low SUDEP risk. Displayed at peak height threshold *T* > 3.2 (*p* < 0.001 uncorrected), with an extent threshold *k* = 110 voxels. Clusters smoothed for display purposes. Covariates included age, sex, weight, and summed intensities of high intensity voxels, ventricular voxels, and white matter voxels. Regions of increased FDG-uptake in patients at high risk of SUDEP are depicted in orange, and regions of decreased FDG-uptake are depicted in blue.

Whole-brain analysis demonstrated that high FBTCS burden—and therefore high SUDEP risk—was associated with a large cluster of increased FDG uptake that included the right cerebellar deep nuclei, cerebellar vermis, pontine tegmentum, dorsal midbrain/PAG, bilateral ventral diencephalon, bilateral thalamus, bilateral pallidum, left putamen, and left claustrum (Figure 1, depicted in orange). These high-risk subjects also showed a cluster of decreased activity in the left planum temporale (Figure 1, depicted in blue).

### Effect of Frequency of FBTCS

**Figure 2:**
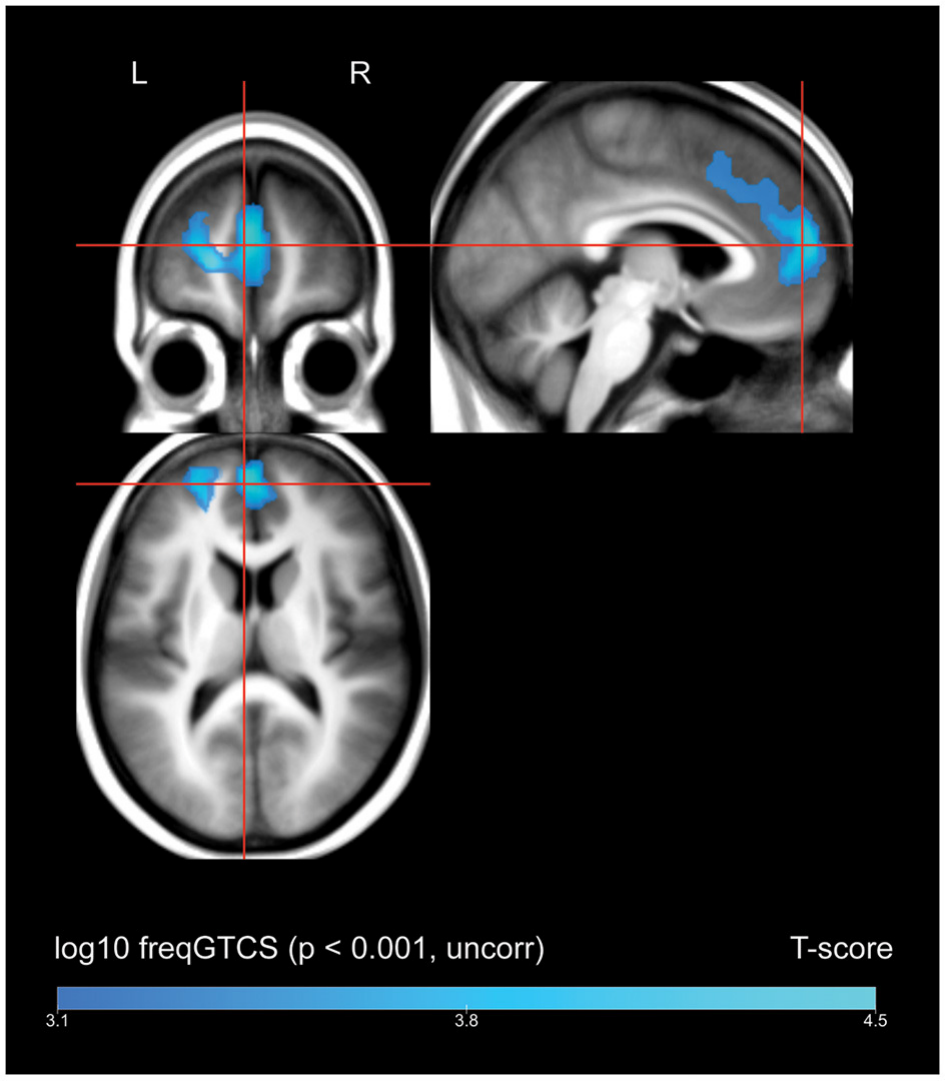
Whole brain SPM results, regression of frequency of FBTCS against FDG-uptake. Displayed at peak height threshold *T* > 3.2 (*p* < 0.001 uncorrected), with an extent threshold *k* = 110 voxels. Clusters smoothed for display purposes. Covariates included age, sex, weight, and summed intensities of high intensity voxels, ventricular voxels, and white matter voxels. Regions of where increasing frequency of FBTCS predicts decreased FDG-uptake are depicted in blue.

A whole-brain regression analysis evaluating the effect of frequency of FBTCS on ^18^FDG uptake found that increasing frequency of FBTCS was associated with a cluster of decreased FDG-uptake in the bilateral medial superior frontal gyrus, with extension including dorsal aspects of the left anterior cingulate gyrus (Figure 2, depicted in blue).

## Discussion

### Overview

Our principal finding is that multiple brain regions known to be involved in cardiovascular, breathing, and aspects of somatomotor regulation show altered metabolism in patients with a relatively high frequency of FBTCS. These patients are known to be at relatively high risk of SUDEP. Increased metabolic activity appeared in the basal ganglia, ventral diencephalon, midbrain, pontine tegmentum, and deep cerebellar structures, as detailed below. To be sure, these regions subserve a vast array of functions. However, the functions that they have in common are related to regulation of cardiorespiratory function, including autonomic regulation, motor initiation and coordination, respiratory timing, integration of hypoxia and hypercarbia with breathing patterns, adrenergic regulation, modulation of awareness, interactions of breathing with blood pressure, and dampening of apnoea and blood pressure extremes. Decreased metabolism appeared in the left planum temporale.

To establish at the overlap between our results and known autonomic regions, we queried the online Neurosynth database (https://neurosynth.org/analyses/terms/autonomic, acquired May 16, 2021). An automated meta-analysis of human functional neuroimaging studies (23), yielded a map of activations more likely to appear in publications referencing the term “autonomic” than those not referencing this term. The resulting map demonstrates activations in nine of the twelve regions identified in the present study—including the right cerebellum, cerebellar vermis, pontine tegmentum, dorsal midbrain/PAG, bilateral ventral diencephalon, left thalamus, left putamen, and left claustrum—and suggests that these regions are consistently implicated in studies of autonomic function. That these regions demonstrate altered metabolism in patients with a high frequency of FBTCS represents an important addition to our understanding of interictal metabolism in patients at the highest risk of SUDEP, which is likely a consequence of centrally-mediated autonomic and cardiorespiratory collapse.

### Volume vs. Metabolic Changes

In previous work, we demonstrated that patients who have died of SUDEP demonstrate decreased gray matter volumes in the posterior thalami, medial and lateral cerebellum, and PAG—regions known to be critical for cardiorespiratory recovery (11, 12). Patients at high-risk of SUDEP—as ascertained by FBTCS frequency—also showed volume loss in the cerebellum and thalamic regions, although to a lesser degree. Disturbance or loss of neuronal elements is typically associated with regional hypometabolism, and in the presence of atrophy, there may be an additional element of artifactual hypometabolism due to partial volume effects (24, 25). At first glance, it may appear surprising that these regions are associated with *increased* FDG-uptake in patients at high risk of SUDEP assessed in this study. However, hypermetabolism has been seen in other conditions marked by loss or dysfunction of neurons. A critical distinction is that glucose uptake does not reflect only neuronal metabolism—under normal circumstances, half of the glucose leaving capillaries is taken up by astrocytes (26). Under neurodegenerative conditions, regional astrocyte and neuroinflammatory cell metabolism may result in areas of relative hypermetabolism (27, 28). In an FDG-PET study of 32 patients with ALS, hypermetabolism appeared in the midbrain and pons, regions in which neurodegeneration of corticospinal neurons is present (29). Subcortical and cerebellar regions of hypermetabolism in a patient with ALS were shown to worsen over 20 months (30), changes that were associated with either an increase, decrease, or no change in cortical thickness, suggesting that atrophy and metabolism need not be coupled. In the present study, areas of increased metabolism may well co-localize with atrophy, and may indicate underlying gliosis and neuronal loss or ongoing inflammatory responses.

Alternatively, the co-occurrence of atrophy and hypermetabolism may suggest an upregulation of neuronal activity. In patients with Alzheimer's disease, a mismatch develops between atrophy and blood flow, with atrophy-corrected cerebral blood flow increased to the hippocampus compared to healthy controls (31). Whether such upregulation may occur in the hypermetabolic regions identified here is not known at this time.

Finally, normalizing PET data to the global mean can result in an artifactual appearance of hypermetabolism in subcortical structures (32). However, in this study, we have not used global normalization, and while the sums of high intensity voxels were included as a covariate, these sums do not differ between the high and low risk groups (Table 2). Furthermore, there is no *a priori* reason to assume that patients at higher risk of SUDEP have lower global mean glucose uptake.

### Increased FDG Uptake in Regions Involved in Autonomic Control

Increased FDG uptake within the left putamen, bilateral pallidum, bilateral thalamus, bilateral ventral diencephalon, and left claustrum may reflect a pathological disturbance that contributes to disrupted autonomic functions, i.e., a profound hypotension or altered sympathetic or parasympathetic drive to the heart, placing patients at risk for SUDEP. The basal ganglia patterns are of principal concern because of their significant roles in autonomic control, and especially blood pressure maintenance (33, 34). The posterior thalamus demonstrates deficient responses to hypoxia in patients with CCHS (35), is especially important for control of hypoxic responses during early development (36, 37), and shows increased metabolic activity up to 2 weeks following prolonged hypoxia (38). This region exhibits decreased volume and diminished functional connectivity with brain stem structures in patients at high risk of SUDEP (12, 13), which raises the possibility that it may fail to respond appropriately to hypoxia in this risk group.

The dorsal striatum helps to modulate blood pressure changes (33) and motor program initiation, where “motor program” includes coordination of the upper airway musculature with the diaphragm. The putamen, in particular, participates in initiation of motor action, and demonstrates profound abnormalities in patients with obstructive sleep apnoea, which may contribute to the failure to activate upper airway muscles in a timely fashion before diaphragmatic descent, leading to airway obstruction in that syndrome (39). Decreased connectivity occurs between the putamen and anterior cingulate cortex, i.e., motor control and autonomic areas in patients at high risk of SUDEP (13), and central apnea respiratory muscle cessation can be induced by blood pressure elevation (40). Changes in putamen/cingulate cortex connectivity may contribute to failure in those interactions.

The ventral diencephalon, which includes the hypothalamus, ventral thalamus, subthalamus, and epithalamus, helps to govern a range of autonomic functions including thermal regulation, and both parasympathetic and sympathetic outflow. This region is extensively damaged in CCHS, obstructive sleep apnoea, and heart failure, all of which are associated with sudden death (41). The ventral diencephalon receives direct inputs from limbic cortical regions via the subiculum and may thus be modulated by seizures (42). Whether the hypothalamus plays a direct role in SUDEP is not yet known, but the structure exerts profound influences on blood pressure; thus, baseline metabolic or structural changes within that area are of concern.

The claustrum is an intriguing region with respect to epilepsy and SUDEP. Situated between the insular cortices and the putamen, the claustrum is involved in sensory integration and consciousness. It has robust connections to almost all cortical areas, as well as subcortical areas including the putamen, globus pallidus, and lateral amygdala (43, 44). The claustrum integrates and modulates widespread neuronal activity, and sustains very focal damage in a subset of cases of refractory status epilepticus (45). There is evidence that the claustrum is a common area involved in ictal and interictal activity across focal epilepsies, and patients with frequent seizures have reduced GABAa receptor binding in this region, as measured by flumenazil-PET (46). With respect to SUDEP risk, altered function of the claustrum may modulate downstream regions involved in autonomic regulation.

A cluster of regions with increased metabolism in patients with a high frequency of FBTCS extended from the midbrain, caudally through the pontine tegmentum, and involved the bilateral cerebellar vermis and right cerebellar deep nuclei. These regions are heavily involved in autonomic and respiratory regulation. Of great relevance for SUDEP, the cerebellar areas serve a “last resort” role to dampen extremes of blood pressure or recover from prolonged apnoea (47–49). Lesions within the cerebellar deep nuclei, for example, will lead to an inability to recover from profound hypotension (48), a significant concern in SUDEP, with a loss of blood pressure in postictal periods providing a circumstance for cardiovascular collapse. The PAG serves critical roles in the control of breathing and the perception of breathlessness (50), and demonstrates decreased volume in patients who have died of SUDEP (11, 51). A recent study demonstrated that when fluoxetine blocks respiratory arrest in an animal model of SUDEP, significantly increased PAG activation results (52), suggesting a potential role in recovery from apnoea. The parabrachial nucleus of the pons, locus coeruleus, and dorsal raphe nucleus are implicated in CO_2_-induced arousal (53), and are damaged in CCHS, another condition with a high prevalence of sudden death (41). These nuclei are also damaged in infants who die of SIDS (5), again suggesting their potential importance to the pathophysiology of SUDEP.

Cerebellar areas project heavily to vestibular and brainstem areas which then integrate with autonomic and respiratory areas of the brainstem. Cerebellar structures, and particularly the vermis, provide essential integration for compensatory responses to hypotension, and beat-to-beat maintenance of cardiovascular homeostasis (54). The cerebellum also modulates breathing in response to hypercapnia, particularly via the fastigial nuclei, which project to pontomedullary nuclei and the posterior thalamus, and cerebellar responses to hypercapnia are impaired in patients with CCHS (35, 55). Cerebellar volume loss appears in many patients with epilepsy, but is most marked in patients who have died of SUDEP, even after controlling for anti-epileptic drug exposure (11). Given this volume loss, the present results suggest that, as noted above with respect to patients with ALS, patients at increased risk of SUDEP may have active inflammation or gliosis in these regions.

### Decreased Interictal FDG Uptake in Left Planum Temporale

A pronounced decline in FDG-uptake in the high FBTCS group was present in the left planum temporale, a region that constitutes part of Wernicke's area, and is implicated in speech and language processing (56). The relevance of these findings is unclear, though regional interictal hypometabolism may reflect areas of overlapping functional deficit zone in our patient group (57).

### FBTCS Frequency as a Proxy for SUDEP Risk

Frequency of FBTCS is the strongest predictor of SUDEP. However, the FDG-PET findings we have reported are directly reflective of the interictal metabolic pattern associated with a propensity to generate FBTCS, and represent an indirect evaluation of SUDEP risk. In this study, two patients died of SUDEP, and both were in the high risk group. As a tertiary referral center engaged in epilepsy surgery evaluations, all of our patients are likely at a higher risk than the general population of people with epilepsy. There is likely a referral bias toward patients with a higher seizure burden, greater number of anti-seizure medications, and refractory epilepsy. Those undergoing FDG-PET are more likely to have MRI-negative focal epilepsy. However, with reference to Table 1, our groups do not differ in terms of epilepsy duration, overall seizure frequency, or number of anti-seizure medications. As such, we are confident that our results reflect a difference between those patients with frequent FBTCS vs. those with none.

It is difficult to disentangle the effects of FBTCS and SUDEP risk. However, our additional regression analysis by FBTCS frequency did not identify any overlapping significant clusters. This finding suggests that the interictal metabolic patterns we reported reflect a difference between patients with a propensity to generate FBTCS and those without. If the effects were driven entirely by the occurrence of FBTCS, we might expect to see the same interictal patterns associated with a greater frequency of FBTCS. This was not the case, which suggests that the metabolic patterns for FBTCS frequency and SUDEP risk, as defined by a propensity to produce FBTCS, may be distinct. This caveat, however, can only be addressed fully if a well-calibrated risk score were available.

We note that our high- and low-risk groups did not differ with respect to number of AEDs or duration of epilepsy. These two factors also emerged as important risk factors for SUDEP (7), and might therefore be expected to differ between our groups. However, this finding likely reflects the referral bias at our center toward patients with drug-resistant epilepsy. Furthermore, the adjusted OR for polytherapy (1.95) and duration of epilepsy (1.95) are less marked than the adjusted OR for ≥3 FBTCS (15.46) (7).

Risk classification could be improved by including physiological data, such as presence of hypoxia, post-ictal generalized EEG suppression, apnea, and/or heart rate variability. It will also be important to attempt to replicate these findings in patients who died of SUDEP relative to patients with epilepsy deemed to be at low risk of SUDEP. These replications could be done in a case-controlled retrospective manner, allowing for an enriched sample.

## Conclusions

High frequency of FBTCS is associated with hypermetabolism in regions of the diencephalon, cerebellum, midbrain, and pons. These findings suggest a possible association between SUDEP risk and functional changes in brain regions that subserve a variety of critical functions, including cardiorespiratory regulation. Whether these alterations reflect compensatory changes or damage is unknown. It is premature to speculate on the processes underlying altered metabolism in these regions that lead to increased SUDEP risk; it does appear, however, that blood pressure regulation and breathing pattern sites, and especially areas underlying recovery from vital challenges are especially targeted.

There are precedents for using PET abnormalities as a biomarker for future events (58). The remarkable metabolic increases found in the basal ganglia, thalamus, brainstem, and cerebellum raise the possibility that these alterations may be useful to predict SUDEP. Considered within the context of structural changes and functional connectivity alterations determined by earlier MRI procedures, these findings shed light on the processes that pre-dispose patients to SUDEP and suggest targets for interventions to avoid neural conditions that lead to fatal outcomes.

## Data Availability Statement

The raw data supporting the conclusions of this article will be made available by the authors, without undue reservation.

## Ethics Statement

The studies involving human participants were reviewed and approved by South West-Central Bristol Ethics Committee. The patients/participants provided their written informed consent to participate in this study.

## Author Contributions

BW prepared and analyzed the data and wrote the manuscript. JW, RH, AJ, BD, FC, SL, and LA advised on interpretation of findings. JW, AJ, and LA advised on imaging analysis. CS advised on clinical and neurophysiological issues. SV, JB, and A-LS advised on imaging and methodological issues. All authors contributed editorially.

## Funding

We are grateful for support from the NIH—National Institute of Neurological Disorders and Stroke (U01-NS090407). BW would like to acknowledge support for his research time through a Samuel R. McLaughlin Fellowship and the University Internal Medicine Research Fund (Dalhousie University, Halifax, Canada), as well as a Detweiler Traveling Fellowship (Royal College of Physicians and Surgeons of Canada, Ottawa, Canada).

## Conflict of Interest

The authors declare that the research was conducted in the absence of any commercial or financial relationships that could be construed as a potential conflict of interest.

## Publisher's Note

All claims expressed in this article are solely those of the authors and do not necessarily represent those of their affiliated organizations, or those of the publisher, the editors and the reviewers. Any product that may be evaluated in this article, or claim that may be made by its manufacturer, is not guaranteed or endorsed by the publisher.
